# Root traits and microbial community interactions in relation to phosphorus availability and acquisition, with particular reference to *Brassica*

**DOI:** 10.3389/fpls.2014.00027

**Published:** 2014-02-11

**Authors:** Paul J. Hunter, Grahams R. Teakle, Gary D. Bending

**Affiliations:** ^1^School of Life Sciences, University of WarwickCoventry, UK; ^2^Warwick Crop Centre, University of WarwickWellesbourne, UK

**Keywords:** rhizodeposition, phosphorus, *Brassica*, rhizosphere, microbial community, root traits, organic acids, phosphatases

## Abstract

*Brassicas* are among the most widely grown and important crops worldwide. Phosphorus (P) is a key mineral element in the growth of all plants and is largely supplied as inorganic rock-phosphate, a dwindling resource, which is likely to be an increasingly significant factor in global agriculture. In order to develop crops which can abstract P from the soil, utilize it more efficiently, require less of it or obtain more from other sources such as soil organic P reservoirs, a detailed understanding the factors that influence P metabolism and cycling in plants and associated soil is required. This review focuses on the current state of understanding of root traits, rhizodeposition and rhizosphere community interaction as it applies to P solubilization and acquisition, with particular reference to *Brassica* species. Physical root characteristics, exudation of organic acids (particularly malate and citrate) and phosphatase enzymes are considered and the potential mechanisms of control of these responses to P deficiency examined. The influence of rhizodeposits on the development of the rhizosphere microbial community is discussed and the specific features of this community in response to P deficiency are considered; specifically production of phosphatases, phytases and phosphonate hydrolases. Finally various potential approaches for improving overall P use efficiency in *Brassica* production are discussed.

## INTRODUCTION

*Brassicas* are one of the most widely grown crops in the world. Worldwide production in 2011 was estimated at between 45 and 60 million tons (Yadav et al., 2011; Agricultural Marketing Resource Centre, 2013), the majority of which was oilseed rape (*Brassica napus*). *B. napus *is primarily grown for oil for human consumption and as feedstock for biofuel (biodiesel) production. The remaining meal is used for animal feed and contains one of the highest concentrations of phosphorus (P) of all crops (15.1 kg t^-^^1^ fresh weight; Potash Development Association). In the UK, over 640,000 ha were under oilseed rape in 2010, with an estimated value of £702 million (UK Agriculture, 2013). Consequently, these crops represent a considerable investment of land and resources, a significant income for farmers, and a substantial component agricultural revenue at national levels.

Phosphorus is a key mineral element in the growth of all plants and is largely supplied in Western agricultural practice as inorganic phosphate (Pi). Widespread P deficiency in soils places serious constraints on plant productivity worldwide (Lynch, 2007). This is either because the soil P concentrations are low or because the P is present in inaccessible forms. In 70% of world agricultural soils, P forms insoluble compounds making it inaccessible to plants. This includes being adsorbed to calcium (Ca), iron (Fe), or aluminum (Al) hydroxides or oxides (Oburger et al., 2011). Approximately 30 million tons of Pi fertilizer is added worldwide to soils each year in order to alleviate this. Rock-phosphate, the source of agricultural Pi is a finite resource that, at current rates of usage, is predicted to become a limiting factor for food production within the next century (Koppelaar and Weikard, 2013). Furthermore, the distribution of the phosphate-bearing rock is extremely localized, potentially giving countries where such deposits occur an effective monopoly over worldwide food production costs and thus food prices. In addition, approximately 80% of agriculturally applied P can also be rendered inaccessible in the same way as naturally occurring P (López-Bucio et al., 2000b), making P fertilization an extremely poor method for delivering P to plants and an inefficient use of this dwindling resource. In order to maintain soil P concentrations in agricultural soils, sufficient P needs to be added back to the soil to replace that removed during cropping. Based on a typical yield of 3.5 t/ha for winter oilseed rape crops (Defra, 2010), this equates to approximately 34,000 tons of P to be replaced in the UK alone. The rendering of considerable proportions of fertilizer P inaccessible to plants however, results in the addition of a large excess of P above the actual plant requirement. Addition of excess P where not required can lead to problems with eutrophication of watercourses, and the growth of algal blooms (White and Hammond, 2009).

**FIGURE 1:**
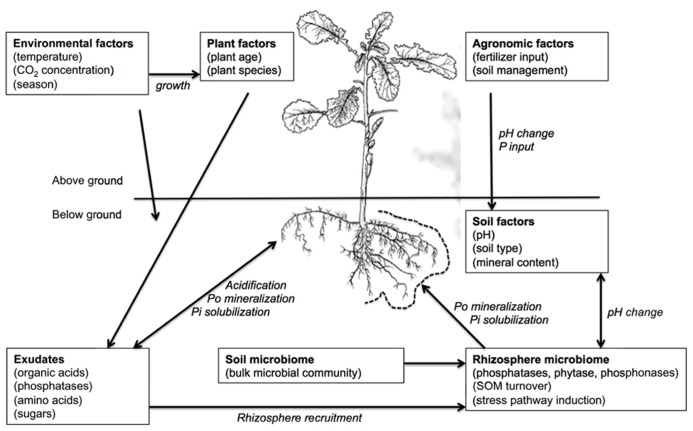
**The various influences on P acquisition of plant root systems and their potential interactions**.

Many factors influence the ability of plants to take up P as Pi from the soil, some of which are interrelated or interacting (**Figure 1**). At the macroscopic scale, agricultural practice, soil amendments (e.g., fertilizer applications) and soil moisture could all impact on P uptake. At the scale of individual plants, the rate of photosynthesis (under the influence of available light and temperature), root system architecture, and the physical properties of the individual roots are able to influence P uptake. Physical root characteristics such as reduced primary root length, thickening of roots, and proliferation of lateral roots and root hairs have been associated with P-deficient conditions (Hammond et al., 2009). The root systems of plants also naturally release a range of compounds from living roots into the surrounding soil in a process called rhizodeposition (Jones et al., 2004). For *Brassica* species, this can account for up to 5% of total photosynthetically fixed carbon (C) under unstressed conditions, with nutrient stresses including low P availability altering both the overall amount of rhizodeposition and the relative proportions of particular components (Shepherd and Davies, 1993). Organic acids are one of the major components of root exudates (Neumann and Romheld, 1999) and these compounds have a significant role in P acquisition in *Brassica* species. Rhizosphere deposits also include a number of exogenous enzymes (Negishi et al., 2002). Key among these for P acquisition, are phosphatases (Hurley et al., 2010), particularly acid phosphatases, which are believed to be responsible for mobilization of organic phosphorus (Po) in the rhizosphere. A number of exudate components are also considered drivers of rhizosphere colonization (Whipps, 2001), with the rhizosphere-colonizing microorganisms subsequently influencing availability of both Pi and Po.

Crops that can abstract P from the soil or utilize it more efficiently, require less of it or obtain P from renewable sources, such as Po reservoirs, need to be developed. In order to facilitate this development, it is imperative to properly understand the mechanisms by which the plant root and its soil environment interact with respect to P acquisition. This review focuses on the current state of understanding of physical and biochemical root traits and concomitant interactions with the rhizosphere microbial community that are involved with the acquisition of P in *Brassica* species and relates those observations to the general paradigms of plant P interactions. While, rhizosphere microbial function is not under direct plant genetic control, plant physical and biochemical characteristics may have considerable influence on the composition and activity of the rhizosphere microbial community. Consequently, plant genotype has an indirect influence on rhizosphere microbial function in regard to P acquisition, allowing for the potential of breeding approaches targeted to both plant- and rhizosphere-based P acquisition pathways. The practicalities and likely pitfalls of various potential strategies for improving overall P efficiency in* Brassica* production are discussed. Further consideration is given to the potential to manipulate the plant rhizosphere composition or function in a controlled manner, either by altering agricultural practices or by modification of the plant exudation profile or by a combination of both approaches.

## ROOT CHARACTERISTICS

Root traits affecting the acquisition of mineral elements will often determine yields in reduced-input agricultural systems (White et al., 2013). Most crop species appear to possess root systems with low tissue densities (Lynch, 2007) and highly branched architectures (White et al., 2005; Lynch, 2007), which are adapted to maximizing nutrient uptake (White et al., 2013). For example, P use efficiency (PUE) is generally correlated with P acquisition efficiency (PAE; White and Hammond, 2008) and with root architectural traits (Hammond et al., 2009), which also correlate with each other (White et al., 2005). A negative correlation was identified between P concentrations and total root length in *Brassica* species; however, within-species variation in *B. napus* indicated that length of exposure to P deficiency is a factor since this relationship was not clearly expressed in younger plants but only in plants that were exposed to low P concentrations up until flowering (Marschner et al., 2007). Phosphorus availability also influences root architectural complexity, i.e., the number, length, density, and growth rate of lateral roots and root hairs in *B. nigra* (Carswell et al., 1996). In addition to total root length (Solaiman et al., 2007), increases in root architectural complexity have been positively correlated with increased PUE (defined as the yield per unit P concentration in plant tissue) in numerous *Brassica *species (Akhtar et al., 2008; Hammond et al., 2009).

Root hairs in *B. napus* increase surface area of the root cylinder by a third (Jungk, 2001). These hairs are responsible for a P uptake rate of approximately 3 fmol cm^-^^1^ s^-^^1^ (a relative high rate among species surveyed: Jungk, 2001). Over 70 quantitative trait loci (QTL) associated with PUE have been detected in *Brassica* with homologs of P transporters from *Arabidopsis thaliana* found to map to the confidence intervals of many of these QTL (Yang et al., 2011). The differential abundance of proteins associated with protein degradation and proteasome activity, the DNA replication cycle (Alexova and Millar, 2013), and actin synthesis and cytoskeletal organization in P stress tolerant and susceptible *Brassica* tissue (Yao et al., 2011), has led to speculation that such changes may indicate a re-allocation of plant resources to root tissues to facilitate the root growth observed in response to P stress (Yao et al., 2011).

Evidence from both *Arabidopsis* (Chevalier et al., 2003) and *B. napus* (Shi et al., 2013) suggests that primary root responsiveness of *Brassicas* to P concentrations may be under genetic control as well as metabolic influence. QTL have been identified for primary root growth responses to low Pi availability (Yang et al., 2010) in *B. napus*. These QTL co-localize with the functional markers BnPHT3-A3 (a trans-membrane P transporter) and the related transcription factor BnWRKY-A3 (Ding et al., 2011), as well as shoot and root dry weight (Yang et al., 2011), root volume and surface area (Yang et al., 2010, 2011), and plant height (Ding et al., 2012). In addition, the locations of these QTL correspond to a region of the *B. oleracea* genome associated with shoot biomass and PUE traits (Hammond et al., 2009). However, the influence of low-P on root architecture may be dependent on the type of root (e.g., lateral root or root hair) and growth stage (formation or elongation; Niu et al., 2013). This may be associated with the fact that expression of some root architecture modifying genes that are induced in response to low P availability are sugar-dependent (Hammond and White, 2011). Root architecture may also be influenced by the release of phytohormones from the rhizosphere microbial communities encountered by the roots (Pitts et al., 1998).

Several plants species (such as *Banksia* or* Protea*) that are naturally adapted to low P environments have developed specialized root structures known as “cluster” root morphology (Grierson, 1992; Denton et al., 2007). These structures increase the root surface area available both for P uptake and the exudation of organic acids (Tomasi et al., 2009). Different plants species have slightly differing root morphologies, but in general, plant responses to P-deficient environments include reduction of the length and thickening of the primary root, proliferation and increased length of lateral roots (Hammond et al., 2004), and increased numbers of root hairs (Daram et al., 1998; Grierson et al., 2001; Hammond et al., 2004). This has a similar effect to the production of cluster roots; an overall increase in root surface area. In addition, a negative correlation between root diameter and rate of root turnover in soil has been determined from a comparison of plant roots from a range of naturally occurring plant communities (Gill and Jackson, 2000). The implication for a potential increase in lifespan of thicker roots compared to finer ones may reflect the potential benefits in nutrient acquisition of such changes in root morphology.

In addition to physical differences, P deficiency has a considerable effect on gene expression, particularly in the roots. For example in tomato plants, P deficiency results in the increased transcription of particular Pi transporter genes that are differentially expressed in root rather than leaf tissues (Daram et al., 1998). Expression of similar genes has been particularly associated with newly formed root hairs in *Arabidopsis* (Mudge et al., 2002) that indicates potentially localized gene expression responses within the root architecture. Many proteins with potential regulatory functions, including post-transcriptional regulation, post-translational modification, and protein degradation, are differentially expressed in *Arabidopsis *under P stress (Alexova and Millar, 2013). While these proteins may represent overarching regulatory functions, more specific differences associated with phenotypes related to differential P acquisition and usage efficiencies have been identified. The differences are potentially related to lateral root growth and involved proteins that are either typically involved in controlling C flow between shoot and root (Chevalier and Rossignol, 2011), or in the mobilization of Po within the plant (Hammond et al., 2004). Furthermore, cellular concentrations of ATP are reduced under P stress (Ueki, 1978), while levels of pyrophosphate are concomitantly increased (Plaxton, 1996), potentially making Po available for re-allocation.

## RHIZODEPOSITION

Rhizodeposition is the release of C compounds from living plant roots into the surrounding soil. This C loss occurs either through passive diffusion, decomposition of roots or sloughed-off root cells or via plant-controlled release, generally in response to nutrient or other environmental stimuli (Jones et al., 2004). Numerous compounds are released as rhizodeposits, including carbohydrates, organic and amino acids, phenolics, fatty acids, sterols, enzymes, vitamins, hormones, nucleosides, polysaccharides, proteins, and phospholipids into the soil, some of which (particularly carboxylic acids) have been particularly associated with P acquisition (Dakora and Phillips, 2002). Due to the nature of many of these compounds (e.g., amino acids, peptides and proteins), rhizodeposition also produces a loss of plant nitrogen (N). However, the secretion of hydrated mucilage (an amalgam of polysaccharides and lipoproteins) forms an interface between the root tip and soil that allows uptake (and exudation) of solutes (reviewed in Carminati and Vetterlein, 2013), which may mitigate some of these losses.

Estimates of the amount of C loss associated with this phenomenon vary and are significantly different in different plant genera, but in general range from approximately 3 to 40% of photosynthetically fixed C, under optimal growing conditions (Gardner et al., 1983; Dinkelaker et al., 1989; Marschner, 2011; Johnson et al., 1996; Grayston et al., 1997; Keerthisinghe et al., 1998; Pinton et al., 2001; Morgan et al., 2005). In *B. napus*, 17–19% of fixed CO_2_ is transported to roots, with 30–34% of this lost to the soil in the form of rhizodeposits, resulting in a net loss of 5–6% of total fixed C (Shepherd and Davies, 1993). In *B. juncea*, the C loss corresponds to approximately 2.8 t C ha^-^^1^ y^-^^1^ (Morgan et al., 2005), which is no longer available for plant biomass and therefore crop yield production. Little information on the specific factors that influence gross rhizodeposition traits in *Brassica* species is available; however, plant age seems to be one such factor. Based on sterile liquid culture experiments, proportions of alanine, glutamine, isoleucine and gamma-aminobutyric acid (GABA) exuded by *B. napus* increased as plants aged, with older plants producing more amino acids per plant, but younger plants having higher concentrations of amino acids per unit mass of root tissue, suggesting a greater overall rate of exudation in younger plants (Shepherd and Davies, 1994).

Both the forms of C and N released from roots and the amounts of rhizodeposition vary depending on plant species (Neumann and Romheld, 1999), nutrient status (Pearse et al., 2006), plant age, root architecture, and environment (reviewed in Curl and Trueglove, 1986; Schilling et al., 1998; Jones et al., 2009; Ryan et al., 2009). However, these factors do not act independently of each other and the interactions may be complex. As observed in *Brassica*, increasing plant age appears generally to decrease levels of rhizodeposition (Nguyen, 2003). The age at which this decrease becomes apparent differs between pot-grown plants and field-grown plants, suggesting an interaction with environmental factors (Meharg and Killham, 1990). Soil C concentrations also increased under elevated CO_2_ in studies on ryegrass (Allard et al., 2006), but no effect was observed when similar CO_2_ concentrations were applied to a mixed grassland plant community (Niklaus et al., 2001). This suggests that different plant species may respond differently to variations in CO_2_ concentrations in terms of rhizodeposition rates. However, in a separate study, under N limited conditions, this type of mixed grassland community did show increased rhizodeposition in response to elevated CO_2_ concentrations (Pendall et al., 2004). Furthermore, plant N status alone (irrespective of CO_2 _ concentrations) has also been found to alter root exudation patterns in barley (Paterson et al., 2006) and *Lolium* (Kuzyakov et al., 2001; Henry et al., 2005). Plant species-specific effects of N on rhizodeposition may be due to variation in the balance between increased fine root growth under higher N concentrations (enhancing rhizodeposition capacity) and a reduction in overall C allocation belowground under N-sufficient conditions (Blagodatskaya et al., 2010). In managed agricultural systems (e.g., *Brassica* production), N deficiency is unlikely to be an issue due to almost ubiquitous nitrogenous fertilizer application, however, as these examples make clear, there is potential for a great deal of variation in overall amount of rhizodeposition. The secretion of these compounds into the soil can also impact directly on the nutritional status of neighboring plants (Schenk, 2006) adding further layers of interaction. While this is unlikely to have a significant impact in monoculture-based agricultural systems, it may be more important in intercropping systems (Bellostas et al., 2003). This cropping strategy has been gaining recognition in some areas of canola production that have previously been under monoculture (Dietz, 2011) as well as in organic farming systems (Bellostas et al., 2003).

### CARBOXYLIC ACIDS

Plant roots typically contain many short-chain organic acids, for example, lactate, acetate, oxalate, succinate, fumarate, malate, citrate, isocitrate, and aconitate. Many of these acids, but particularly malic acid and citric acid (or anionic malate and citrate) have been associated with P mobilization. Malic and citric acid are the most prevalent and abundant organic acids detected in root exudates (Neumann and Romheld, 1999). Furthermore, different cultivars or lines within plant species (including *Brassica* species) that are more or less efficient in P uptake, show differences in the concentrations of organic acids (particularly malate and citrate) in their root exudates (Kirk et al., 1999; Shahbaz et al., 2006; Corrales et al., 2007; Aziz et al., 2011). Additionally, these compounds increase in proportion in the root exudates of many plant species under P deficiency, with such increases often being significantly greater in P uptake-efficient lines compared to inefficient ones (Dinkelaker et al., 1989; Hoffland et al., 1989; Ae et al., 1990; López-Bucio et al., 2000a; Dechassa and Schenk, 2004; Yan et al., 2004; Miller, 2005; Shahbaz et al., 2006). Other organic acids have also been reported to be associated with P-uptake efficiency, although less frequently and often in specific plant species, for example oxalate in *Banksia* sp. (Denton et al., 2007).

#### Carboxylic acid exudation

Basal rates of root exudation are controlled by passive diffusion rates. Phosphorus deficiency can potentially result in increased permeability of the plasma membrane of root cells due to reduction in phospholipid content of the plasma membrane and concomitant increased rates of diffusion of solutes from roots to the soil (Ratnayake et al., 1978). This would result in higher rates of passive solute accumulation in the rhizosphere under P-deficient conditions, without requiring an active plant response (Jones, 1998). However, there is evidence that C efflux can also be directly up-regulated to help alleviate stress (Jones, 1998), e.g., under P deficiency (Lambers et al., 2002). In *B. napus, *increased activities of malate dehydrogenase and phospho-enoyl pyruvate carboxylase/oxygenase (PEPC) were associated with increased rates of citrate and malate release in response to constitutive expression of a bacterial citrate synthase gene (Wang et al., 2013). In addition, P stress has been associated with differential expression of sucrose phosphate synthetase, malate, pyruvate and succinate dehydrogenases, and ATP synthase (Yao et al., 2011). Although much of this increase in activity is believed to derive from *de novo* protein synthesis (Hammond et al., 2004; Wang et al., 2013), increases in activity of pre-existing enzymes, via phosphorylation or other mechanisms, cannot be discounted (Hoffland et al., 1989; Ryan et al., 1995; Johnson et al., 1996). Furthermore, different pathways may potentially control activity levels of some enzymes in different plant species or under different environmental conditions. For example, transcription of PEPC in response to P deficiency has been shown to be up-regulated in some studies on *B. napus* (Duff et al., 1989; Hoffland et al., 1989), but not in others (Wang et al., 2013), and down-regulated in the related plant *Arabidopsis *(Wu et al., 2003). It is conceivable that the activity of this enzyme may be non-transcriptionally regulated. Both malate inhibition and phosphorylation activation of PEPC have been proposed as potential mechanisms (Moraes and Plaxton, 2000), and it seems probable that either or both of these mechanisms may be involved in the regulation of PEPC activity in response to P deficiency.

In some cases, P deficiency alone was unable to induce organic acid exudation in *B. napus*, although reduced plant growth was observed as a consequence of poor P nutrition. Exudation of organic acids (particularly malate and citrate) and activity of a number of enzymes involved in or peripheral to the tricarboxylic acid (TCA) cycle and associated with organic acid exudation (citrate synthase, malate dehydrogenase, and PEPC) were found to be enhanced in response to increased Al concentrations and that this corresponded with reduced cellular metabolism of citrate and malate (Ligaba et al., 2004). Additional evidence for the involvement of Al comes from over-expression of an *Arabidopsis *homolog of citrate synthase in *B. napus*, which resulted in increases in enzyme activity, root-associated citrate levels and tolerance to Al (Anoop et al., 2003). A gene (*TaALMT1*) from wheat (*Triticum aestivum*) roots, which putatively codes for an ion channel-forming protein has been shown to mediate Al-induced malate efflux (Delhaize et al., 2007). Expression of two root-specific *TaALMT1* homologs from* B. napus* (*BnALMT1* and *BnALMT2*)**in heterologous plant expression systems (including *Nicotiana tabacum*) indicated Al-induced malate efflux enhancement from roots. Enhanced efflux was also induced by the presence of a number of other trivalent phytotoxic metal cations (La, Yt, Er) but not by P deficiency. The effect of Al on organic acid exudation was weaker and shorter-lived under P-deficient conditions than under P-sufficient conditions (Ligaba et al., 2006), suggesting that P in some form (potentially ATP as an energy source/phosphorylation activator) is involved in increased exudation of organic acids. It is worth noting that auxin-responsive proteins are differently expressed in P stress-tolerant and stress-susceptible *B. napus* (Yao et al., 2011). Although no direct link has been demonstrated between auxin-responsive proteins and rhizosphere acidification, such proteins may putatively affect activity of plasma membrane proton pumps (McQueen-Mason et al., 1992; Shen et al., 2006).

Although organic acids are exuded from roots, their primary role is as intermediaries in the TCA cycle upon which all cells depend for oxidative energy production. PEPC, malate dehydrogenase, and citrate synthase are involved in the up-regulation of organic acid exudation in many species, with the activities of the enzymes generally increasing in response to P deficiency (Raghothama, 1999). Other enzymes, including RNases, intracellular acid phosphatases, and enzymes potentially involved with respiration and energy production (e.g., mitochondrial aconitase, malic enzyme, alcohol dehydrogenase, and monodehydroascorbate reductase), are differentially expressed in response to P deficiency in *Arabidopsis* and may also contribute to increases in organic acid exudation (Chevalier and Rossignol, 2011). No evidence of differential abundance of any of these enzymes has yet been reported in *Brassica* (Alexova and Millar, 2013).

#### Modes of action of carboxylic acids

Exuded organic acids may potentially influence Pi availability in various ways. Phosphorus is readily adsorbed to soil particulates, particularly oxides or hydroxides of Fe and Al (in acid soils). In this case, chelation appears to be the major mechanism for P solubilization by organic acids under P-deficient conditions (Oburger et al., 2011), and there is evidence from rice for increased P solubilization due to direct chelation of P by exuded citrate (Kirk et al., 1999). Alternatively, organic acids could conceivably either compete with P for the adsorption sites on the soil particles and so reduce the availability of sites for P adsorption, or could actively displace adsorbed P (Jones et al., 2004). Chelation by organic acids also appears to play a major role in the detoxification of trivalent Al ions (by sequestration; Ryan et al., 2009). In addition to Al, citrate has also been shown to chelate Mn, Zn, and Ca ions (Dessureault-Rompré et al., 2008). The chelation of Ca may be particularly important in alkaline soils, where P is often rendered insoluble due to sorption to Ca. Therefore, organic acid solubilization of P may occur irrespective of soil pH conditions, however the organic acid profiles may be different under differing conditions; for example, soil pH influences the ratio of malate to citrate in root exudates from both lupin and ryegrass (Veneklaas et al., 2003). In addition, acidic soil conditions promote acetic acid exudation, while alkaline/calciferous soils promote oxalic and citric acid exudation in many plant species, including *B. napus* (Ström et al., 1994; Zhang et al., 1997). Studies have shown that citrate is more efficient than malate, oxalate, or malonate, at solubilizing P (Earl et al., 1979; Furrer and Stumm, 1986; Jones and Darrah, 1994; Lan et al., 1995; Jones et al., 1996a; Jones and Brassington, 1998; Oburger et al., 2011). This is most likely because the three carboxyl groups in citrate allow for the formation of more stable complexes than the other (di-carboxylic) acids, allowing more efficient complexing of Al in P-bearing rock (Furrer and Stumm, 1986; Jones et al., 1996a), release of adsorbed P from soil matrices (Earl et al., 1979; Lan et al., 1995) or blocking of adsorption sites within the soil matrix (Oburger et al., 2011). However, exudation of organic acids may not always be sufficient to solubilize complexed P: Pea (*Pisum sativum*) and Chickpea (*Cicer arietinum*) are reportedly unable to access Al- or Fe-complexed P, despite exuded organic acid profiles containing relatively high proportions of citrate (Pearse et al., 2007). It should also be noted that the relative efficiencies of the different organic acids in solubilizing P are heavily dependent on soil conditions (Oburger et al., 2011). While increased organic acid exudation can be considered a generally conserved response to P deficiency stress, it is not a ubiquitous response: for example, hedge mustard, a *Brassicacea* of the genus *Sisymbrium, *is reported not to secrete organic acids of any type in response to P deficiency (Hoffland et al., 1989) and it is not certain that a P-deficient environment is the (only) trigger for increased amounts of organic acids in root exudates. Citric and malic acid deposition may also be increased in response to other stressors such as deficiency of other nutrients, including K, Mn, and Fe (Jones et al., 2003b) and the presence of toxic Al (Ligaba et al., 2006). In general however, a small number of organic acid compounds tend to be conserved components within exudate profiles, although considerable variation in both absolute and relative abundances of these compounds occurs in different species and under different environmental stimuli.

The organic acid component of exudates need not directly interact with P in order to influence plant P nutrition however. Organic acids, particularly citric and malic acids, have been associated with decreases in soil pH in response to P-deficient conditions in several plant species (Grierson, 1992; Zhang et al., 1997), including *Brassica* (Raghothama, 1999), with some suggestion that malate may be of more importance in P acquisition than citrate in *B. napus* (Hoffland et al., 1989). This soil acidification is believed to improve P dissociation from mineral sources and promote mineralization of organic forms of P (Neumann and Romheld, 1999). Since organic acids are believed to exist in plant root cytoplasm predominantly in the anionic (dissociated) state (Jones and Brassington, 1998), they would be unlikely to lower soil pH directly, upon exudation. In order to maintain electrochemical balance in root cells, the exudation of such anions would require either influx of other anions, or the exudation of positively charged counter ions. These are generally assumed to be protons (H^+^; Loss et al., 1993; Marschner, 2011), which would result in direct acidification of the rhizosphere soil, although there is some evidence from *Arabidopsis* that potassium (K^+^) may be the counter ion in some cases (Murphy et al., 1999). In* Phaseolus* beans, QTL for both P uptake efficiency and total acid exudation co-localized with QTL for proton exudation (Yan et al., 2004) and an apparent link between citrate exudation and proton efflux from roots via plasma membrane proton-ATPases has been reported in lupin (Tomasi et al., 2009). There is also evidence for the involvement of anion-channel proteins and multidrug and toxin extrusion (MATE)-type proteins (proton anti-porters) in organic acid exudation (Vance et al., 2003). There is little evidence to distinguish between the biological importance of any of these potential mechanisms for rhizosphere acidification and it is possible that the overall pH effect may be a balance between some or all of these potential activities.

Much of the evidence for these potential rhizosphere acidification mechanisms and for the desorption/release of P described above, involve organic acid concentrations 10- to 100-fold greater than the 1–50 μM concentrations typically found in the soil solution (Earl et al., 1979; Lopez-Hernandez et al., 1986; Jones and Darrah, 1994; Baziramakenga et al., 1995; Lan et al., 1995; Krzyszowska et al., 1996; Strobel, 2001). This may be a consequence of the fact that carboxylic acids, particularly malate and citrate, are strongly adsorbed to soil particulates (Jones and Edwards, 1998) and rapidly metabolized by soil microbial populations. Such populations can remove approximately 15 nmol malate kg^-^^1^ soil s^-^^1^ (Jones and Darrah, 1994; Jones et al., 1996b). However, at least one analysis comparing the organic acid concentrations in root sap and soil, suggests that the majority of the rhizosphere organic acid pool comes from a source other than plant roots (e.g., microbial release or as secondary products from the breakdown of more complex precursors; Jones et al., 2003a). The combination of microbial degradation and/or production of organic acids and the potential of organic acids for adsorption to soil particles, mean that any exuded organic acid is unlikely to migrate far from the site of release. This suggests that P solubilization may occur at discrete sites in the rhizosphere, or may be facilitated by different mechanisms at different sites, dependent on the prevailing soil conditions and microbial community. In addition, different regions of the root structure may be associated with different aspects of P acquisition. For example, the exudation of organic acids in *Brassica* species has been particularly associated with root tips, with approximately threefold greater increases in exudation of malate and citrate from these regions than from older root tissue on exposure to low P environments (Hoffland et al., 1989). Phosphate uptake meanwhile has been particularly associated with root hairs (Daram et al., 1998; Hammond et al., 2004; Yan et al., 2004), which do not form at the root tip but in slightly older tissue behind the advancing tip. This is likely to give rise to differences in metabolic activity, including exudate composition and nutrient uptake rates along the length of the roots. Furthermore, root material of the same class (e.g., root tips, lateral roots, root hairs, etc.) from the same plant will inhabit different portions of the soil and thus experience different microenvironments and resulting stimuli. Taken together, these observations suggest that there may be localized P mobilization occurring at discrete sites in the rhizosphere, rather than a uniform level of activity.

While organic acid chelation and rhizosphere acidification are of particular importance in the solubilization and acquisition of Pi by plants, other sources of P, such as soil organic matter (SOM), are also available to plants. Organic phosphorus may comprise up to 65% of the total P in soils (Harrison, 1987). The majority of this soil Po is in the form of chemically stable inositol phosphates (e.g., phytate) and phosphonates, with a smaller portion present in the form of orthophosphate esters, and organic polyphosphates (Turner et al., 2002).

### PHOSPHATASES

Enzymes secreted by both plant roots and soil microorganisms mediate Po mineralization processes. Among the key plant enzymes involved in P acquisition are exogenous phosphatases, particularly acid phosphatases and phytases (Duff et al., 1994; Ryan et al., 2009). These enzymes are among the exudate components actively secreted in response to P-deficient environments (Negishi et al., 2002), and are thought to be responsible for mobilization of Po in the rhizosphere by releasing P from organic compounds (Ahokas and Manninen, 2001; Vance et al., 2003; Hammond et al., 2004; Hurley et al., 2010).

Phosphatases are a class of enzymes that are excreted both by plant roots and components of the microbial community. In *B. napus*, acid phosphatase activity is significantly greater in P deficiency tolerant lines than in susceptible lines irrespective of P conditions (Zhang et al., 2009), although P deficiency induction of root-secreted acid phosphatase activity has also been demonstrated (Zhang et al., 2010). Orthologs of purple acid phosphatase (an Fe-metalloprotein) from *Arabidopsis* have been mapped to intervals spanned by QTL in *B. napus* that were associated with P uptake and usage efficiency (Yang et al., 2011), and transcription of these genes can be induced in response to P deficiency (Lu et al., 2008). Certain acid phosphatases in *B. napus* may also be indirectly regulated by PEPC, the enzyme potentially associated with organic acid exudation in response to P deficiency. In *B. napus*, PEPC activity is increased by glucose-6-phosphate and reduced by malate, aspartate, glutamate, and isocitrate (Moraes and Plaxton, 2000). Increases in the activity of PEPC in response to medium-term (days) exposure of plants to a P-deficient environment appear to be due to increased transcription, rather than alteration in the specific activity of the enzyme (Moraes and Plaxton, 2000).

Plant-derived phosphatase activity is known to be associated with the root tips of many plants, including maize (Dinkelaker and Marschner, 1992) and potato (Zimmermann et al., 2004). It was also strongly expressed along the root axis of potato, although not significantly in root hairs. Phosphatase activity has also been associated with the specialized cluster root structures of plants adapted to P-deficient environments (Denton et al., 2007). These differences may indicate alternative mechanisms for P acquisition, which allow the plant to utilize parts of the root system other than root tips and root hairs, where the majority of the exudation of organic acids associated with P uptake occurs. There is also some evidence to suggest that exogenous phosphatase activity is associated with the root mucilage (a combination of polysaccharides, phospholipids and proteins), which is actively secreted from growing root cap (Jones et al., 2009).

One of the major sources of soil Po is phytate, which reportedly comprises between 40% and 80% of the soil Po (Steffens et al., 2010; Mukhametzyanova et al., 2012), although it is thought to only comprise a very small proportion of residual plant material (e.g., <1% by mass of plant stem material returned to cropped soils; Steffens et al., 2010; Mukhametzyanova et al., 2012; Noack et al., 2012). This suggests either a very high content of returned plant stem material in soil or an alternate source of phytate. Proportions of phytate are at least 45 times higher in seeds than stems (Noack et al., 2012), where it is used as a C and P reserve for emerging seedlings. It is possible that the soil Po estimates include phytate from soil seed banks. However, it is unlikely that such reserves of phytate would be directly accessible, even to plants that exude phytases. For example, transgenic plants with increased secretion of microbial phytases showed comparable growth and had similar P status to control plants in many soils (George et al., 2004; George et al., 2005). Such seed-based reserves would likely require breakdown of seeds or emerging seedlings by microbial pathogens or necrotrophs before the phytate became available for P mineralization. Phytate also forms the majority of the Po component of manure and slurry (Hill et al., 2007), along with phospholipids and nucleic acids (Turner and Leytem, 2004). This form of phytate is likely to be far more directly accessible, however, there is little evidence for plant-derived phytase activity in the rhizosphere. Very little root exuded phytase activity has been found in plants (Richardson et al., 2000; Ponstein et al., 2002) and where present, activity is low. For example, in wheat root exudates, phytase activity was at least 20 times lower than that of exogenous phosphatase (Richardson et al., 2000). This suggests that the majority of rhizosphere phytase activity is likely to come from another source (e.g., the soil microbial component). In addition, both phytate and phytase are tightly adsorbed to many of the mineral components of soil. Phytate is rendered unavailable for dephosphorylation by such adsorption, while soil phytase activity declines by up to 95% over a 24-h period. The exact rate of this decline and the retention of adsorbed enzyme on soil particulates are dependent on the soil mineral composition (Giaveno et al., 2010), although there is evidence that the presence of total SOM, which may include phytate, mitigates sorption-based inhibition of phytase activity. This suggests that directly plant-available phytate may be considerably less than the total phytate soil pool and indicates a significant role for the soil microbial community in influencing P (particularly Po) availability.

## RHIZOSPHERE MICROBIAL COMMUNITY

In addition to nutrient deficiencies, root exudate profiles also respond to other stimuli including salicylic and jasmonic acids, and chitosans (Walker et al., 2003), which form part of the signaling cascade in plant defense responses, including the systemic acquired resistance (SAR) pathways. Responses include the exudation of a range of secondary metabolites, for example phyto-alexins (phenolic defense compounds produced in response to pathogens; Brigham et al., 1999; Flores et al., 1999) and a number of allelopathic compounds (released by one plant that affect the growth or development of another; Hirsch et al., 2003). Some general plant defense response pathways, such as SAR, can be induced by the colonization of roots by non-pathogenic bacteria (De Meyer et al., 1999). Inoculation of hydroponically grown *B. rapa* with either zoonotic pathogens or with the human gut commensal organism and common soil bacterium *Bacillus subtilis* (none of which are plant pathogens) for example, produced significant increases in amounts of phenolic compounds in the leaves (Jahangir et al., 2008). The metabolic components induced differed depending on the bacterial species inoculated, however in general, Gram-positive bacteria induced increases in the amounts of fumarate, the cellular signaling molecule gamma-aminobutyric acid and coumaroyl-malate (a phenolic derivative of malate) in leaves, while Gram-negative bacteria increased the amounts of two other malate-based phenolic acids in the leaves (Jahangir et al., 2008). Plant defense responses to microbial colonization of the rhizosphere may be of particular importance in P metabolism as it has been suggested that plant responses to P deficiency may be regulated via general stress response pathways as well as through P-specific responses (Hammond et al., 2004). More recent evidence of the association of a number of elements involved in general stress–response pathways, such as ethylene biosynthesis (Thibaud et al., 2010), reactive oxygen species, and transcriptional repressors (Niu et al., 2013), with P acquisition would seem to support this suggestion. Furthermore, proteins such as chaperonins, receptor-like kinases, auxin-response proteins, protein modification, and glycolytic enzymes, proteins involved in cell wall lignification, oxidative stress responses and secondary metabolite production (i.e., involved in general stress responses) were differentially expressed in P stress tolerant and P stress susceptible lines of *Brassica* (Yao et al., 2011). In addition, certain phenolic compounds can catalyze release of Po, making it available for both the microbial and plant communities (Jones et al., 2009). The fact that both plant-pathogenic and non-pathogenic micro-organisms can potentially influence P availability via the plant root system highlights the importance of the rhizosphere microbial community in soil nutrient cycling and on plant growth and nutrient acquisition.

### RHIZOSPHERE MICROBIAL RECRUITMENT

Almost all plant-derived exudates can act as C and N sources to some portion of the microbial community and consequently may function as microbial attractants (Dakora and Phillips, 2002; Matilla et al., 2007). Many soil-borne organisms follow gradients of plant exudate components, including sugars and amino acids, toward the root (Whipps, 2001). This leads to a proliferation of microorganisms within the different compartments (endo-rhizosphere, rhizoplane, and ecto-rhizosphere) under the umbrella term “rhizosphere” (Barber, 1995), which can be defined as the soil environment that is influenced by the presence and activities of roots (Vanpeer and Schippers, 1989). The rhizosphere supports microbial populations up to 20-fold denser than detected in the surrounding bulk soil (Lynch, 1987; Lynch and Whipps, 1990; Bending, 2003; Morgan et al., 2005) although it is less diverse (Marilley et al., 1998; Marilley and Aragno, 1999). Organic acids, which have been strongly implicated in P solubilization and acquisition, are also considered key drivers in bacterial chemotaxis from bulk soil to the rhizosphere (Jones et al., 2003a). For example, variation in citric acid concentrations have been implicated in differences in both bacterial and fungal community structure, with the bacterial community also responding to variation in the concentrations of *cis*-aconitic and malic acids (Marschner et al., 2002). Root-associated microbial populations also tend to concentrate near features of root architecture associated with rhizodeposition, including the root tip and root hairs (Ramos et al., 2000). Several studies have also demonstrated differences in rhizosphere communities between root zones (Yang and Crowley, 2000; Duineveld et al., 2001; Marschner et al., 2002). In addition to organic acids, glucose, which is the most common sugar in rhizodeposits (Derrien et al., 2004), significantly increased both soil microbial activity (measured as biomass increase) and microbial phosphatase activity (Spohn et al., 2013). The amino acid alanine had a similar effect but more pronounced than that of glucose, although the lag phase between organic input and change in activity was longer than for glucose. The differences are likely because alanine provides a source of both C and N, rather than simply C (as in the case of glucose), but also requires prior metabolism before it can be utilized, while glucose provides C in a directly accessible form (Spohn et al., 2013). Many soil microbes are also able to utilize organic acid-complexed P or uncomplexed acids (Kucey et al., 1989; Whitelaw, 2000; Richardson, 2001; Vessey et al., 2004). This makes them potential competitors for the plant produced resources and solubilized P. However, although P was incorporated in soil microbial biomass early on in response to organic addendums to the soil, the increased amounts of P in microbial biomass were not maintained (Spohn et al., 2013).

### RHIZOSPHERE MICROBIAL ACTIVITY

Rhizosphere microorganisms are thought to stimulate the dissolution of insoluble minerals from soil in a way similar to that proposed for plants exudates (Jahangir et al., 2008). Many rhizosphere bacteria, particularly from the genera *Pseudomonas, Burkholderia, Enterobacter, Bacillus,* and *Citrobacter*, some actinomycetes and mycorrhizal fungi, are capable of general enhancement of P solubilization and/or the release of P and other minerals from rocks (reviewed in Vessey, 2003; Hoffland et al., 2004; Puente et al., 2004; Barea et al., 2005; Poonguzhali et al., 2006). In addition to potentially competing for Pi, many components of the rhizosphere microbial population are able to access Po. Mycorrhizal fungi comprise one of the most intensively studied groups of rhizosphere microorganisms. Over 2,000 fungal species are capable of forming mycorrhizal communities (arbuscular and ecto-mycorrhizae), with some ecto-mycorrhizal fungi able to produce a suite of extracellular enzymes that mobilize organic forms of N and P (Read and Perez-Moreno, 2003). Although *Brassica* species are considered to be non-mycorrhizal, root exudates (particularly breakdown products of glucosinolates) of several *Brassicaceae* have been shown to be able to stimulate spore germination of a number of ecto-mycorrhizal fungi (Zeng et al., 2003). In addition, glucosinolates are known to influence microbial community composition when contained in *Brassica* seed meal soil amendments and have been associated with increased abundance of organisms associated with fungal disease suppression (Hollister et al., 2013).

Pseudomonads (β-proteobacteria) are common colonizers of plant tissues in general, including roots (Espinosa-Urgel, 2004), and have traditionally been considered to be major components of many rhizosphere communities (Marilley and Aragno, 1999; Lugtenberg et al., 2001; Yang et al., 2001), including those from *Brassica* roots, which were predominantly colonized by proteobacteria (particularly *Pseudomonads*), actinobacteria, and *Bacteroidetes*. Although *Pseudomonas* remain a major component of the rhizosphere community in *B. napus*, other microbial groups may dominate, for example *Bacillus* spp. (Macrae et al., 2001; Poonguzhali et al., 2006). The precise microbial components associated specifically with P release/solubilization are generally not known and are likely to vary depending on environmental conditions and other influences. Factors known to influence the composition of the rhizosphere community include soil type (Marschner et al., 2001), which determines the bulk soil pool from which the rhizosphere community can be recruited, and plant age (Smalla et al., 2001). However, the composition of rhizosphere communities are largely determined by the plant species with which they are associated (Smalla et al., 2001), primarily by selection for organisms capable of utilizing the C source profile produced by the roots (Grayston et al., 1998; Grayston et al., 2001). Rhizosphere microbial communities are also subject to successional change in response to changing plant and environmental stimuli (Yang and Crowley, 2000). Furthermore, multiple members of the community may carry out similar microbial functions (functional redundancy) and the capacity to carry out such functions may be mobilized between individual community members. Horizontal plasmid transfer, for example, has long been shown to occur at elevated levels in the rhizosphere (for a review see van Elsas et al., 2003).

One of the key groups of enzymes involved are the phytases. Phytase production has been identified in isolates of numerous bacterial and fungal genera associated with P solubilization (Richardson and Hadobas, 1997; Kim et al., 2002; Hill et al., 2007; Daynes et al., 2008; Ghorbani-Nasrabadi et al., 2012; Mukhametzyanova et al., 2012; Miao et al., 2013). Identification of acid and alkaline phosphatase activities has been limited to the common soil-borne genera *Pseudomonas* and *Enterobacter* (Krey et al., 2011)*, Bacillus *(Ramesh et al., 2011; Yadav and Tarafdar, 2012; Hu et al., 2013) and *Aspergillus* (Tarafdar and Yadav, 2011), with isolates of *Rhizobium *(Jonas et al., 2008)*, Bacillus *and *Pseudomonas* (Khushi et al., 1993) identified as producing phosphonate hydrolases. These latter enzymes, which may be controlled by both P-inducible and P-independent regulatory mechanisms (Quinn et al., 2007), are related to acid phosphatases but mineralize Po in the form of phosphonates. Phosphonates are strongly adsorbed to mineral particles (Nowack and Stone, 2006) and extremely stable due to the presence of a direct C–P bond, rather than the more usual and easily hydrolyzed phospho-ester (P–O–C) bond (Kim et al., 2011). This makes them a highly inaccessible source of Po, the importance of which is only just beginning to be understood.

In addition to the plant influence on the composition of the microbial community, plants may influence community activity. Many soil micro-organisms are dormant in the absence of organic input due to C limitations on growth (Joergensen et al., 1990). There is evidence that the growth of plants in soil is associated with an increased rate of turnover of SOM in the order of two- to threefold (Cheng, 2009). The organic input associated with growing plants comes predominantly from rhizodeposition, although the degradation of dead roots with concomitant cycling of nutrients may also contribute (Kuzyakov, 2010). Rhizodeposition produces a pulse of organic input that often generates hotspots of microbial activity and associated SOM turnover around areas of exudation. The nature of this distribution and the limitations of current investigative techniques mean that there is a dilution effect involved in the measurement of the SOM turnover. Based on a notional 10% of the rhizosphere involved in producing hotspots, this equates to an actual increase of SOM turnover in the order of 20- to 30-fold in the hotspots themselves (Kuzyakov, 2010). Within the hotspots, microbial turnover is believed to increase, in addition to SOM turnover. Bacterial r-strategists (fast growing species that utilize simple substrates) rapidly metabolize easily accessible organic material resulting in increased biomass. These organisms are then replaced by species with the capacity to degrade and utilize more complex substrates, but with relatively slow growth (k-strategists; Andrews and Harris, 1986; Brimecombe et al., 2001). These are likely to be fungi, particularly since they are able to grow though low nutrient zones (i.e., between hotspots) by means of hyphal extension (Otten et al., 2001), although Gram-negative bacteria have also been implicated (Nottingham et al., 2009). It is possible that these secondary colonizers can benefit from the turnover of the microbial biomass of the r-specialists as the easily utilized C sources are depleted (Fontaine et al., 2003). This turnover of the microbial biomass also makes P indirectly available to the plant roots (Oehl et al., 2001), in addition to any release of Po from the SOM breakdown. These hotspots have a lifetime of a few days (Pausch and Kuzyakov, 2011), suggesting that soil nutrient availability may vary over relatively short temporal periods in localized regions of the rhizosphere.

Rhizosphere microbial populations are not spectators; individual species and organisms interact both with other members of the communities and with the plant roots. Plant–microbial interactions can be broadly classified as pathogenic, neutral (where no benefit or harm to either partner is involved), positive (where either one partner derive benefits from the association without harming the other), or symbiotic (where both partners benefit). From a practical standpoint, neutral interactions are often difficult to assess due to lack of suitable measures (Paterson, 2003) and are unlikely to influence microbial population dynamics significantly. However, pathogenicity, positive, and symbiotic interactions will have significant influences on both plant and rhizosphere microbial community. Furthermore, interactions between plant roots and soil organisms can influence adjacent plants (Li et al., 2007). For example, homoserine lactone, a degradation product of the bacterial regulatory signaling molecule *N*-acyl-homoserine lactone, increases stomatal conductance, and transpiration (Joseph and Phillips, 2003), potentially influencing plant water and nutrient status. Taken together this represents a highly complex and evolving picture of the relationship between plants and rhizosphere microbial communities with respect to P cycling. The ubiquitous nature of the soil microbial component in plant production systems, and the potential impacts of microbial activity on many other factors such as root morphology and plant chemistry that influence both Pi and Po availability and acquisition, make rhizosphere microbiology one of the key areas in understanding P dynamics in agricultural systems.

## FUTURE PERSPECTIVES FOR IMPROVING P ACQUISITION IN *BRASSICA* CROPS

The decreasing availability and increasing cost of P fertilizer is likely to be an increasingly significant factor in much of global agriculture in the next few decades, not just for *Brassicas*. In order to continue to meet the food production demands of the world’s population, the agriculture sector will need to find methods of crop production that either require less P or make better use of the existing P reservoirs in soils. The fact that *Brassica* crops, and oilseed rape in particular, are a major contributor to agricultural economies, and the high levels of genetic synteny between commercial *Brassica* crops and the model plant *Arabidopsis*, mean that there is already a considerable knowledge base and available research resources associated with this crop.

Changes in root growth patterns and redistribution of resources to support increased root growth are associated with P deficiency. The changes not only increase the root surface area available to acquire Pi directly, but also increase the density and distribution of root zones exuding rhizodeposits. In *Brassica*, these rhizodeposits can account for considerable proportions of fixed C and the exudation of certain organic acids (malate and citrate) are known to increase under P deficiency (although increases in these compounds may also be responding to Al^3^^+^, particularly associated with mineral complexed Pi in acidic soils). In addition, differential abundance of enzymes associated with the TCA cycle (i.e., sucrose phosphate synthetase, a number of TCA cycle-intermediate dehydrogenases and ATP synthase) have been associated with P stress (Yao et al., 2011). Plant roots also release enzymes, particularly acid phosphatases, with other enzymes that are considered important in the mineralization of Po (phytases, alkaline phosphatases, and phosphonases), appearing to be primarily microbial in origin (Quinn et al., 2007; Richardson et al., 2009; Tarafdar and Yadav, 2011; Mukhametzyanova et al., 2012). Based on existing knowledge, the potential routes toward reducing P input in* Brassica* crop production can be described broadly as manipulation of root architecture, organic acid release, Po cycling, rhizosphere microbial interactions, and P utilization, although all are interrelated to some extent and can be considered under the umbrella term of rhizosphere engineering (Ryan et al., 2009).

### PHOSPHATE ACQUISITION

Root architecture appears to be associated either directly or indirectly with many aspects of P acquisition (Carswell et al., 1996; Solaiman et al., 2007; Akhtar et al., 2008; Hammond et al., 2009) making it an obvious target for the development of crop plants with enhanced P acquisition ability. Furthermore, in *B. napus*, numerous root architectural traits have been shown to be heritable, indicating underlying genetic control mechanisms that have the potential to be exploited in breeding programs (Shi et al., 2013). A number of QTL associated with such traits appear to be conserved within the *Brassicaceae* (White et al., 2013), allowing for potential cross-species exploitation of genetic information derived both from within this group of important crop plants and *Arabidopsis*. In terms of altering root architecture, increasing the spread of lateral roots [particularly in the topsoil region where the majority of Pi is located in most soils (Lynch and Brown, 2001)], and selecting for traits that increase root surface area, e.g., thicker roots and/or increased number, length, and density of root hairs are all potential plant breeding targets. Root hairs have one of the greatest potentials for enhancing P acquisition relative to the cost of production to the plant, making them key targets for breeding programs (Brown et al., 2013), particularly with respect to alterations to root hair length (Jungk, 2001) and longevity (Lynch and Ho, 2005). However, despite clear phenotypic variations by which to easily screen individuals, many of these traits appear to be under complex genetic control with large environmental interactions (Lynch, 2007).

Plant breeding or genetic manipulation approaches can also be focused either on increasing organic acid biosynthesis or on enhancing transport and export of the organic acid anions to the rhizosphere. The organic acids most strongly associated with P acquisition in *B. napu*s, malate, and citrate, are key components of the TCA cycle (Lowenstein, 1969). This is the key regulatory process involved in cellular energy production. Consequently, manipulating concentrations of either of these substrates artificially has potential for unintended consequences that could impinge on many other functions at cellular, organ, and whole plant levels. Manipulation of transport and/or exudation functions might be a more promising target, particularly as work already carried out has identified two families of trans-membrane transporter protein families (MATE and ALMT; Delhaize et al., 2007), of which the ALMT type has already been targeted in *B. napus* (Ligaba et al., 2006). Improving resistance to toxic trivalent metals, particularly Al (which is also associated with organic acid exudation and ALMT transporters), may also be of indirect benefit by allowing for better root growth (Ligaba et al., 2006) and thus increasing the root area available for P uptake. In addition to targeting organic ion export functions, proton transporters, and mechanisms of indirect soil pH alteration might also make suitable targets, as would P import mechanisms such as the Pht1 family of Pi stress inducible Pi transporters (Mudge et al., 2002).

### TARGETING SOIL ORGANIC PHOSPHORUS

Organic forms of P constitute a significant component of the total soil P pool, but are generally considered unavailable for direct plant uptake. Organic P is mineralized to Pi by the action of enzymes, in particular, phosphatases and phytases, which can be of either plant or microbial origin (Adams and Pate, 1992), or microbial phosphonate hydrolases. Increasing either rates of production and release of plant forms of these enzymes or increasing their activity (or both) provides additional targets for breeding or genetic manipulation programs. In many crop species, the presence of mycorrhizal fungi associated with the roots also help to improve P uptake compared to non-mycorrhizal plants. *Brassica* species are not considered mycorrhizal (Smith and Read, 1997); consequently this avenue for microbial mediated P uptake is unavailable, although there is some evidence that AM fungi may be able to colonize canola roots in the absence of other micro-organisms (Marschner and Timonen, 2004). More fundamental work on the nature of the plant–mycorrhizal interaction may identify key plant factors that influence mycorrhizal colonization, opening up the potential for mycorrhizal *Brassicas* to be developed in the longer term. There is some tentative evidence from *Arabidopsis* that suggest plants may be able to take up Po directly from the soil environment: *Arabidopsis* can grow on nucleic acids as the sole P source (Chen et al., 2000; Richardson et al., 2000) and oligonucleotides of up to 25 nucleotides in length have been identified, intact, within *Arabidopsis* roots (Paungfoo-Lonhienne et al., 2010), suggesting direct uptake and allowing for at least the potential for subsequent utilization of the incorporated Po. Further investigation of the potential for direct Po uptake (including investigation of the range and limitation of potential sources of Po) is warranted and the high level of genetic synteny between *Arabidopsis* and *Brassica* species means the results from the model system may be directly relevant to agricultural crop development programs.

#### Rhizosphere manipulation

In addition to mycorrhizal fungi, many other soil microorganisms are also capable of mineralizing Po (Richardson et al., 2009; Tarafdar and Yadav, 2011; Mukhametzyanova et al., 2012). To be effective as an inoculant, a microbial strain must be able to establish and persist in a range of soil types and conditions, and among the resident microbial communities present in those soils (Ryan et al., 2009). While a general increase in Po mineralization in soils may increase available Pi for plant uptake, plants are likely to derive maximum benefit if the Pi is released in close proximity to the roots; i.e., within the rhizosphere (Oburger et al., 2011). In this case the inoculated organism must additionally be capable of recruitment to the rhizosphere and of establishing and persisting in this more heavily colonized environment (Morgan et al., 2005). The development of microorganisms as seed dressings in a similar manner to that which can be used to deliver rhizobia to legume crops (Deaker et al., 2003), would alleviate the need for active rhizosphere recruitment, by delivering the inoculant directly to the emerging radicle, however this would not eliminate the need to establish and persist in a complex microbial environment. Furthermore, the inoculant would still be required to deliver the desired function efficiently in this new environment. Given that specific inoculants are likely to respond differently to different chemical and microbial environments present in the soil, and may only be appropriate for specific plant species, the impracticalities of producing suitable microbial inoculants to improve Pi availability becomes clear. One alternative may be to consider using microbial consortia as inocula. This potentially increases the range of environments in which the inoculum can function and/or the range of plant species for which it is appropriate, but introduces additional variables such as the requirement of the components of the consortium to be able to co-exist with each other.

Another approach would be to use the plants themselves to manipulate and engineer the naturally occurring soil microbial community. Modern, high throughput, metagenomic, and transcriptomic platforms allow community level evaluation of interactions between plants and their microbial environments to a much greater depth than ever before. Plant transcriptomic studies have the potential to identify genes that are associated with recruitment of specific microorganisms, including those known to enhance P availability. Furthermore, meta-transcriptome analysis of rhizosphere and soil communities has the potential to highlight functional rather than compositional variations in microbial communities Thus it may be possible to identify components of plant genomes associated with recruitment of specific microbial functions to the rhizosphere rather than specific microorganisms. If identified, such genes could then be used as targets in breeding programs; potentially enhancing the range of environments the plant can gain benefit from. Rhizosphere manipulation approaches (at least in any controlled manner) remain speculative, but the technologies have now been developed to a point at which it is at least feasible to consider examining the complex interactions involved in appropriate detail.

The use of different agricultural practices could also be adopted to manipulate rhizosphere composition or function. Changes in soil pH, soil compaction, irrigation levels, crop rotation, and land management strategies all have potential to impact on the soil microbial community. While there have been some studies considering P uptake and availability associated with these characteristics (reviewed in Pierzynski and Logan, 1993; Ulen et al., 2010; Li et al., 2011; Balemi and Negisho, 2012), high throughput molecular platforms should allow for much more in-depth investigations of the impact of land management strategies and agricultural practices on soil ecosystems and functions. For example, even relatively simple decisions, such as the form of N fertilizer applied, has the potential to significantly affect P acquisition. The supply of N to plant roots in the form of ammonium tends to cause rhizosphere acidification, whereas nitrate application tends to cause alkalization (Shen et al., 2011), with a concomitant effect on the rhizosphere microbial community and soil pH; a major factor influencing the availability and acquisition of Pi and the mineralization of Po. Another example is subsoil compaction. This usually occurs on agricultural land from the use of heavy agricultural machinery and can limit plant root spread and hence access to water and mineral elements (Whalley et al., 2006; Valentine et al., 2012).

#### P use by plants

Finally, in addition to releasing more Pi from the existing soil reservoirs, plants which utilize P more efficiently will ultimately require less Pi fertilizer input and potentially allow the soil P reservoirs to last longer. Many non-agricultural plants that are adapted to low P soils have relatively low internal P concentrations. This seems to be offset by relatively high specific rates of photosynthesis (i.e., rates of photosynthesis per unit leaf area; Lambers et al., 2010). While this mechanism appears beneficial on the surface, from a crop plant perspective it would be unhelpful, since it appears to be associated with a high leaf mass to leaf area ratio and reduced overall plant growth rate (Lambers and Poorter, 1992). Further detailed investigation of the biochemistry and genetics underlying these effects, however, may allow for the undesirable associations to be broken. Another potential target for altering P use is the ability of plants to reduce the phospholipid concentration in membranes to some degree under P-limited conditions (Essigmann et al., 1998). Studies have shown that plants naturally decrease phospholipid content in thylakoid membranes and in root tissues under P stress (Peñaloza et al., 2002; Raven, 2013). Two of the key phospholipases involved in this process have been identified (Li et al., 2006) and could represent potential targets for breeding programs. During this process, plants replace the phospholipids with galactolipids and/or sulpholipids (Raven, 2013) and potential targets for investigation have been identified in the galactolipid production pathway of the *Brassica*-related model plant *Arabidopsis *(Härtel and Benning, 2000). In grain crops, including cereals, legumes, and oilseed rape, there is also theoretically the opportunity to reduce the P content of the grains. Phosphorus is stored in seeds largely in the form of phytate. Phytate comprises a considerably higher proportion of seed mass in oil-yielding plants (e.g., *B. napus*, linseed, and sesame) than many other plant species, with proportions in *B. napus* as much as 2.5% of seed mass and accounting for approximately 1% of plant P (Lott et al., 2000). These amounts are similar to those for seeds of other oil-yielding plants (linseed and sesame) and are considerably higher than any other seeds (Lott et al., 2000). Strategies to minimize this component of seeds may reduce the requirement for P input. Production of grains with reduced P content in crops used for human or animal consumption could have significant implications for nutritional quality of grains and may impact on plant vigor. Phytate is known to have anti-nutritional characteristics, strongly binding important divalent mineral cations such as Ca, Mg, Fe, Cu, and Zn (Cheryan and Rackis, 1980). Reduction of this component of seeds would therefore have additional nutritional benefits. Studies in rice have also indicated that grains of this crop produced with lower levels of P do not suffer adverse effects on seedling vigor (Rose et al., 2010, 2012).

### AGRICULTURAL PRACTICE

In addition to reducing the amount of P abstracted from the soil, increasing the amount of P returned to the soil pool following cropping would be beneficial. This would involve increasing the proportion of P in the uncropped plant organs. In the case of *B. napus*, this would be the roots and stem and leaf material from non-fodder crops. The practice of conservation tillage (leaving plant debris from the previous crop on the soil surface during and after tillage of the next crop) for example, would not only return more P to the soil but has also been reported to increase soil P solubility (Zibilske and Bradford, 2003). This would be particularly beneficial if combined with increased soil capacity to mineralize organic Po.

### ROUTES FORWARD

Routes to attaining many of these goals are likely to ultimately converge on the generation of new crop cultivars and varieties. Given the two timescales involved; depletion of worldwide P stocks within decades at the current rate of usage and the length of time it takes for plant breeding programs to deliver useable varieties (in the order of 10–15 years from identification of candidate genes and suitable markers), the immediacy of this work is obvious. Modern crop varieties have been systematically bred for better yield (arrived at through many avenues, e.g., improved disease resistance, better physical attributes, etc.). The introgression of even a single characteristic into that highly tuned genetic background is fraught with difficulties and many avenues may prove fruitless. Since P plays such a significant role in plant metabolism, selection for better yield may have inadvertently optimized much of the genotypic variation associated with P metabolism. The consequences of this are that any improvements may well be incremental rather than step-changes in P efficiency. As a result, it may be that in order to achieve the overall aim of reducing P usage, many approaches may have to be deployed simultaneously. This represents a huge program of research followed by an equally large development program. Even with modern marker-assisted breeding techniques, the development time of new varieties cannot be shortened much further. The use of GM technologies offers a potential way to shorten the time required to develop such new varieties, particularly in light of the highly complex genetics that may well be involved. Ultimately the decision on the use of GM crops rests with public acceptance or otherwise. Despite uptake of some GM technologies in China and North and South America (Johnson et al., 2007), the EU moratorium on GM crops remains in place – perhaps it is time to re-open the debate, although this may require a shift in approach (Johnson et al., 2007).

## Conflict of Interest Statement

The authors declare that the research was conducted in the absence of any commercial or financial relationships that could be construed as a potential conflict of interest.
